# Effect of perceived HIV risk on initiation of antiretroviral therapy during the universal test and treat era in South Africa

**DOI:** 10.1186/s12879-021-06689-1

**Published:** 2021-09-20

**Authors:** Sabina M. Govere, Sean Galagan, Boikhutso Tlou, Tivani Mashamba-Thompson, Ingrid V. Bassett, Paul K. Drain

**Affiliations:** 1 Discipline of Public Health Medicine, School of Nursing and Public Health, University of KwaZulu-Natal, Durban, South Africa; 2 AIDS Healthcare Foundation, 162 ZweMadlala Road, Section W, Umlazi, Durban, 4041 South Africa; 3 School of Medicine, University of Washington, Seattle, USA; 4 Faculty of Health Sciences, Prinshof Campus, University of Pretoria, Pretoria, South Africa; 5 Department of Global Health, University of Washington, Seattle, USA; 6 Division of Infectious Diseases, Massachusetts General Hospital, Boston, USA; 7 Medical Practice Evaluation Center, Massachusetts General Hospital, Boston, USA; 8 Center for AIDS Research, CFAR, Harvard University, Boston, USA; 9 Harvard Medical School, Boston, USA

**Keywords:** Perceived risk, Universal test and treat, Rapid ART initiation, HIV/AIDS, 90–90–90, Retention in care

## Abstract

**Background:**

South Africa has not achieved the 90–90–90 goals, in part due to low rates of antiretroviral therapy (ART) initiation among those aware of their HIV status. Perceived risk of HIV at the time of testing may affect likelihood of rapid ART initiation. The purpose of this study was to evaluate factors associated with perceived risk of HIV and the relationship between perceived HIV risk and rapid ART initiation during the universal test and treat era which was adapted in October 2016.

**Methods:**

We conducted a prospective study of adults undergoing HIV testing from October 2016–February 2019 at Ithembalabantu Clinic in Durban. Eligible participants reported not previously being diagnosed with HIV. Before HIV testing, participants were asked to assess their perceived HIV risk on a four-level scale. We categorized “definitely not” and “probably not going to acquire HIV” as a low perceived risk, and “probably will” and “definitely will become HIV-infected” as a high perceived risk of HIV infection. Participants were followed for up to 14 months following HIV testing to assess ART initiation.

**Results:**

Among 1519 people newly diagnosed with HIV, 55% were female and mean age was 33 years. Among those, 1382 (90.9%) had a high HIV risk perception and 137 (9.1%) reported low HIV risk perception. In the low risk group individuals were more likely to be female (58% vs 55%), unemployed (62% vs 59%), have a partner with unknown HIV status (61% vs 55%) compared to the high risk group. 83.2% of those with low HIV risk perception reported previously HIV testing compared 91.5% of those with high HIV risk perception. In the multivariate model, males were associated with a higher chances of initiating ART compared to females (adjusted hazard ratio (aHR): 1.187, CI 1.187 (1.060–1.329) and being unemployed (aHR 0.767 CI (0.650–0.905). Those with a low HIV risk perception were less likely to initiate ART 125 (91%) vs 1310 (95%) p = 0.022), and took longer to initiate on ART after HIV diagnosis (11 days’ vs 4 days, p = 0.042).

**Conclusion:**

Factors associated with high HIV risk perception included being unemployed, single, and having a partner of unknown HIV status. People living with HIV (PLHIV) in South Africa who had a low self-perceived risk to HIV infection were less likely to initiate ART. Assessing self-perceived risk of HIV infection may help direct counselling and improve ART initiation to achieve universal 90–90–90 goal.

## Background

Anti-retroviral therapy (ART) has transformed HIV/AIDS from a severe communicable disease with high mortality to a controllable chronic condition for which early treatment initiation is considered vital [1]. The UNAIDS “95–95–95” treatment strategy recommends that by 2030, 95% of people living with HIV will be diagnosed, 95% of those diagnosed will be on ART, and 95% of those on ART will be viral suppressed [2]. WHO strongly recommends ART initiation on the same day as HIV diagnosis based on the person’s willingness and readiness to start ART immediately, unless there are clinical reasons to delay treatment under the universal test and treat programme (UTT) [3, 4].

South Africa adopted the UTT strategy in 2016 and this led to increased testing efforts, however, ART initiation within the expected period remains a problem [5]. Various testing strategies to reach the first 90 have been adopted by South Africa, including door to door, index and moonlight testing. However, linkage to care in the expected timeline remains a challenge [6, 7], hence high ongoing HIV transmission [8]. Ameliorating factors causing delay in presentation to HIV care and ART initiation are fundamental in ensuring success of HIV programmes [9]. Beliefs around perceived risk to HIV infection are crucial in understanding what influences people to test and later initiate on treatment in the event of a positive diagnosis [10]. An individual who perceives they are susceptible to HIV infection sees themselves as being at risk [11].

People come to test with different perceptions of their risk of getting infected or not getting infected [12]. We hypothesized that people with high perceived risk of HIV infection are more likely to commence ART immediately as part of UTT services and that delayed treatment seeking behaviours are associated with low perceived risk. Denial contributes to commencing treatment late and poor adherence to ART when it is commenced [13]. Association among perceived risk to HIV infection, sexual risk behaviours and treatment adherence has been described in previous studies [13, 14]. However, there is limited knowledge on the factors associated with HIV perceived risk and rapid ART initiation. The purpose of the study is twofold: first to determine factors associated with perceived risk of HIV and, second, the relationship between perceived HIV risk with rapid ART initiation and retention in care in Umlazi, an urban township of Durban in the Kwazulu-Natal province of South Africa.

## Methods

### Study design

We conducted a prospective cohort study of adults (18 years and older) seeking HIV counselling and testing at Ithembalabantu Clinic in Umlazi township between October 2016 to February 2019. The study was approved by the University of Washington’s Institutional Review Board (#49563) and the University of KwaZulu-Natal’s Biomedical Research Ethics Committee (#BF052/13). All methods were carried out in accordance with relevant guidelines and regulations according to the ethical boards.

### Study population

We included adults seeking voluntary HIV testing and counselling with a new HIV-positive diagnosis. Pregnant women and individuals who were not ART naïve were excluded from the analysis.

A research assistant consented all the participants and written informed consent was also obtained from legal guardians of participants having No Primary schooling in the study. Baseline information including demographics, history of HIV testing and prior ART use was collected. Assessment of perceived risk of HIV infection was performed prior to HIV testing using a four-level scale, the levels being: definitely not going to acquire HIV, probably not going to acquire HIV, probably will become HIV-infected and definitely will become HIV-infected [15, 16]. Subsequently a non-research trained HIV test counsellor conducted an HIV test according to the South African guidelines [17].

### Outcome definitions

The main outcomes were perceived HIV risk and immediate uptake of ART. This study differentiates rapid ART initiation and late ART initiation. We defined ART initiation as the day HIV medication is dispensed to the individual and expected to start taking them. In this paper the date of HIV diagnosis minus date of ART initiation defines time to ART initiation and 1 day refers to the definition for rapid ART initiation. Retention in care was categorised as still in care, transferred out, defaulter/loss to follow up and deceased. In our study, participants were classified as still in care if they have been in contact with the facility within the last 3 months. Transferred out participants are those that for any reason request to seek care at another facility and defaulter/LTFU if he/she had not had contact with the clinic for 3 months or more since their last recorded expected return date. Participants were classified as deceased if confirmed by next of kin and using the South African Home affairs website which identifies death status at the date and time of the enquiry.

### Statistical analysis

We employed the Statistical Package for the Social Sciences (SPSS) version 25 for data analysis. A p-value of less than 0.05 was deemed statistically significant. We compared factors associated with low and high HIV risk perception using Chi^2^ tests. We used univariate and multivariate cox proportional hazards modelling to determine the association between HIV risk perception and other demographic variables of interest with time to ART initiation from HIV testing.

## Results

### Cohort characteristics

Of the 3156 participants enrolled, 1519 (48%) were HIV-infected and met the inclusion criteria. Within the study cohort 841 (55.4) were female with a mean age of 33 years, 849 (55.9) had completed high school, majority of 1421 (93.5) were never married (single) (Table 1). About half of the cohort was unemployed 905 (59.6). The majority of the participants 854 (55.8) had a partner with unknown HIV status. 1378 (90.7%) reported previously testing for HIV (Table 1).

**Table 1:** Sociodemographic characteristics of study population

Variable	Frequency (N = 1519) n (%)
Gender	
Male	678 (44.6)
Female	841 (55.4)
Age in years, mean (SD)	33 (9.4)
Education	
No primary school	12 (0.8)
Primary school	39 (2.6)
Some high school	576 (37.9)
Completed high school	849 (55.9)
Higher degree (university)	43 (2.8)
Marital status	
Married	78 (5.1)
Never married (single)	1421 (93.5)
Widowed/divorced	20 (1.3)
Currently employed	
No	905 (59.6)
Yes, working ≤ 20 h per week	313 (20.6)
Yes, working > 20 h per week	301 (19.8)
Partner HIV status	
Partner HIV status unknown	854 (55.8)
Partner HIV negative	277 (18.4)
Partner HIV positive	388 (25.8)
Previously tested for HIV	
Yes	1378 (90.7)
No	141 (9.3)
Perceived risk to HIV infection	
Definitely not going to acquire HIV	22 (1.4)
Probably not going to acquire HIV	115 (7.6)
Probably will become HIV-infected	317 (20.9)
Definitely will become HIV-infected	1065 (70.1)

### Demographic associations with HIV risk perception

**Fig. 1:**
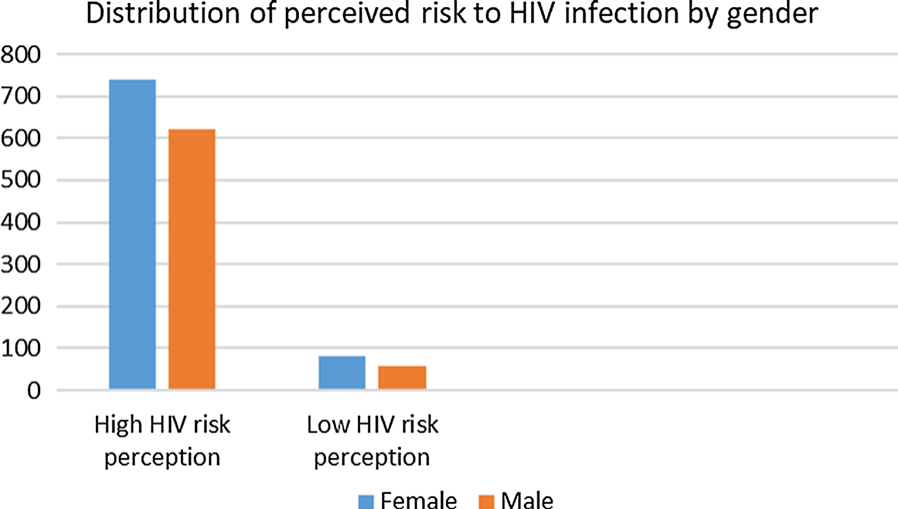
Association of perceived risk to HIV infection and gender

Of the 1519 participants who met the eligibility criteria 1382 (90.9%) participants had high HIV risk perception and 137 (9.1%) reported low HIV risk perception. Participants with high HIV risk perception were more likely to be female (55% versus 41%, p = 0.025) (Fig. 1), to have not completed matric (40% versus 21%, p = 0.008), to be unemployed (79% versus 3%, p = 0.023) and to be never married (97% versus 86%, p = 0.013) (Table 2). There was a significant difference in previous HIV testing with 83.2% of those with low HIV risk perception reported previously been tested for HIV compared to 91.5% of those with high HIV risk perception (p = 0.002).

**Table 2:** Demographic associations with perceived risk to HIV infection

Variable	High HIV risk perception^a^ N = 1382 n (%) or n (Mean ± SD)	Low HIV risk perception^b^ N = 137 n (%) or n (Mean ± SD)	p-value
Gender			
Males	621 (44.9)	57 (41.6)	0.025
Females	761 (55.1)	80 (58.4)	
Education			
No matric	569 (41.2)	58 (43.3)	0.025
Completed high school (matric)	771 (55.8)	78 (56.0)	
Higher degree (university)	42 (3.0)	1 (0.7)	
Employment status			
Not employed	820 (59.3)	85 (62.0)	0.024
Yes, working ≤ 20 h per week	279 (20.2)	34 (24.8)	
Yes, working > 20 h per week	283 (20.5)	18 (13.2)	
Marital status			
Married	69 (5.0)	9 (6.5)	0.027
Never married	1295(93.7)	126 (92.0)	
Widowed/divorced	18 (1.3)	2 (1.5)	
Previously tested for HIV			
Yes	1264 (91.5)	114 (83.2)	0.002
No	118 (8.5)	23 (16.8)	
Partner HIV status: n (%)			
Positive	373 (26.9)	15 (10.9)	0.001
Negative	239 (17.3)	38 (27.8)	
Unknown	770 (55.8)	84 (61.3)	

^a^Participant reported the belief that they probably or definitely will be infected with HIV

^b^Participant reported the belief that they probably or definitely will not be infected with HIV

### HIV risk perception and clinical outcomes

Participants with high perceived HIV risk were diagnosed with lower CD4 count (mean 361 versus 507) compared to those with low HIV risk perception. There was a strong association between HIV risk perception and rapid ART initiation as those with high HIV risk perception initiated ART in an average of 4 days (standard deviation [SD]: 3.67) compared to 11 days (SD: 40.91 days) in those with low HIV risk perception (p = 0.027) (Table 3). Participants in the high HIV risk perception group were more likely to be retained in care compared to the low HIV risk perception group 1039 (79.1) versus 23 (29.5) p = 0.001. There was a strong association between lost to clinical follow-up and low HIV risk perception 33 (42.4) p = 0027.

**Table 3:** ART initiation and clinical associations with perceived risk to HIV infection

Variable	High HIV risk perception^a^ N = 1382 n (%) or n (Mean ± SD)	Low HIV risk perception^b^ N = 137 n (%) or n (Mean ± SD)	p-value
Baseline CD4 count (cells/mm^3^)	361 (242.3)	507 (260.2)	0.027
Initiated ART within 12 months of enrolment	1310 (95.1)	125 (91.2)	0.022
Time to ART initiation (days)	4 (3.67)	11 (40.91)	0.042
Participant status at end of clinical follow-up:			
Retained in care at study clinic	1039 (79.1)	23 (29.5)	0.001
Transferred to another HIV clinic	17 (1.4)	17 (21.7)	0.637
Mortality	61 (4.6)	5 (6.4)	0.447
Lost to clinical follow-up	197 (14.9)	33 (42.4)	0.027

^a^Participant reported the belief that they probably or definitely will be infected with HIV

^b^Participant reported the belief that they probably or definitely will not be infected with HIV

### Rapid ART initiation

Univariate Cox proportional hazards modelling indicated a significant association between demographic variables and rapid to ART initiation including having a previous HIV test (hazard ratio [HR]: 0.819, 95% confidence interval [CI] 0.684–0.981), male gender (HR: 1.262, CI 1.136–1.402) being unemployed (HR: 0.749, 95% CI 0.637–0.881) (Table 4). Partner HIV status, marital status, age, HIV risk perception and education level were not significantly associated with rapid to ART initiation. In the multivariate adjusted model, male gender was associated initiating ART more rapidly compared to female gender (adjusted HR (aHR): 1.187, CI 1.187 (1.060–1.329) as was unemployment compared to employment more than 20 h a week (aHR: 0.767, CI 0.650–0.905). Controlling for other demographic factors, there was no association between rapid to ART initiation and HIV risk perception (aHR: 1.00, CI 0.73–1.37) (Table 4).

**Table 4:** Unadjusted and adjusted odds of ART initiation by sociodemographic characteristics

Demographic factors	Unadjusted (N = 1504)		Adjusted (N = 1519)	
	HR (CI)	p-value	aHR (CI)	p-value
Previous HIV test				
Yes	1.0	Ref	1.0	Ref
No	0.819 (0.684, 0.981)	**0.030**	0.897 (0.742,1.085)	0.264
HIV risk perception				
Low HIV risk perception	1.0	Ref	1.0	Ref
High HIV risk perception	1.014 (0.0845, 1.216)	0.885	1.027 (0.885, 1.217)	0.882
Gender				
Females	1.0	Ref	1.0	Ref
Males	1.262 (1.136, 1.402)	**0.001**	1.187 (1.060,1.329)	**0.003**
Age (continuous)	0.996 (0.991, 1.002)	0.158	0.997 (0.991, 1.003)	0.326
Current employment				
Employed more than 20 h	1.0	Ref	1.0	Ref
Employed for less than 20 h	0.953 (0.834, 1.089)	0.479	0.930 (0.810, 1.068)	0.304
Unemployed	0.749 (0.637, 0.881)	**0.001**	0.767 (0.650, 0.905)	**0.002**
Education level				
Higher degree	1.0	Ref	1.0	Ref
None (primary school was not completed)	0.647 (0.330, 1.268)	0.204	0.730 (0.371, 1.436)	0.361
Primary school	0.929 (0.597, 1.446)	0.929	1.007 (0.639, 1.588)	0.976
Some high school (but not matric)	0.952 (0.695, 1.304)	0.759	0.983 (0.715, 1.352)	0.916
Matric (completed high school)	0.974 (0.714, 1.329)	0.869	1.013 (0.714, 1.384)	0.936
Marital status				
Widowed/divorced	1.0	Ref	1.0	Ref
Married	0.787 (0.475, 1.304)	0.353	0.948 (0.554, 1.623)	0.847
Never married (single)	0.820 (0.521, 1.291)	0.391	0.912 (0.557, 1.494)	0.715
Partner HIV status				
Yes, HIV positive	1.0	Ref	1.0	Ref
No or unknown status	1.099 (0.971, 1.243)	0.134	1.070 (0.940, 1.219)	0.304
Yes, HIV negative	1.050 (0.897, 1.230)	0.543	1.057 (0.899, 1.244)	0.502

The bold figures represents variables which were statistically significant

## Discussion

In this cohort of people newly diagnosed with HIV in South Africa, people who reported high HIV risk perception before their HIV test were significantly more likely to have a positive HIV diagnosis than individuals who reported low HIV risk perception. This reveals that most of the individuals who came to Ithembalabantu Clinic for testing were expecting a positive HIV diagnosis, considering that it is a clinic focused exclusively on HIV treatment management. High HIV risk perception was significantly associated with female gender, and was only moderately associated with age. Other factors associated with high HIV risk perception included being unemployed, being single, and having a partner of unknown HIV status and having an HIV positive partner.

Studies that have examined demographic factors associated with high perceived risk to HIV have found younger age, female gender, low socioeconomic status, unemployment, and marital status, as predictive of greater risk to HIV infection [10]. We found young adults, females, those who were unmarried, those who didn’t know their partner’s HIV status and unemployment to be associated with high perceived risk to HIV infection.

The CD4 count in the high HIV risk perception group was low at 361 cells/mm^3^ showing that the majority presented at the clinic when they were already symptomatic or with relatively advanced disease. The uptake of ART was faster among those with a high HIV risk perception, taking them an average of 4 days to initiate on ART compared to 11 days for the low risk perception group. This shows the successes of universal test and treat and linkage in HIV treatment programs. This is in accordance with the 2015 WHO recommendations for treatment as prevention measure in initiating individuals who test positive for HIV to be initiated immediately on ART [18].

Most studies have explored the association between perceived risk and risk behaviour. High perceived risk has been shown to be associated with high risk behaviour [19]. A study in Malawi found that lack of knowledge impacts negatively on risk perception [20, 21]. To our knowledge, our study was the first to explore the association of perceived risk for HIV infection with rapid/immediate initiation on ART, in a large cohort of South Africans presenting for HIV testing. Our findings indicate healthcare workers, especially those involved with HIV counselling and testing, should intensify pre HIV counselling as well as offer continuous counselling services to ensure that those with low HIV risk perception can accept their results in the event of a HIV positive result and accept immediate ART initiation. New research questions can be identified to improve uptake and efficiency of the current UTT policy.

Participants with no high school and those with matric perceived themselves more at risk compared to those with degrees. Increase in educational level raises awareness which makes individuals to recognize exposure to risk, further supported by the decrease in risk perceptions to HIV infection in people with degrees having more information to allow them to make informed decisions. Our data suggested that unemployment is one of the factors contributing to exposure in risk taking behaviours, as shown by the high perceived risk to HIV infection of the unemployed group. There was a positive association between marital status and perceived risk, people who were single had high perceived risk compared to their married or widowed/divorced counterparts. Community prevention programmes may still improve awareness levels in these high-risk groups. This is in accordance with the WHO recommendations for HIV prevention [22].

We hypothesized that people with high perceived risk of HIV infection are more likely to commence ART immediately as part of UTT services, which might be due to increased acceptance and advanced expectations of a positive result. Reduced fear and stigma coupled with willingness to disclose HIV status to their family or the community might also motivate individuals to seek treatment in a timely manner [23]. Those who had high HIV risk perception were more likely to start ART rapidly compared to those with a low HIV risk perception. The clinical appearance and CD4 count of individuals are important factors for HIV that may prevent or drive timely treatment initiation. We found strong evidence of association between ART initiation and CD4 count in our study and significant association between high perceived risk and immediate ART initiation. Individuals who had a high perceived risk took fewer days to initiate ART compared to those with low perceived risk to HIV. Retention in care of participants with high HIV risk perception and those with low HIV risk perception was moderately different. Of the 11% participants in the low HIV perceived risk group, 75% remained in care similarly closer to 69% retention in care in the high HIV perceived risk group. This shows that in as much as individuals accept ART, remaining in care and defaulting to treatment in still a challenge in HIV treatment management.

Retention in HIV care is important for optimal treatment outcomes and effective positive prevention. Though it is expected that each ART facility keep a record of whether all “transfer out” patients are “transferred in”; the published data on whether the transferred out patients really reach another health care facility is scanty. It is highly possible that some patients recorded as loss to follow up might be unreported transfers out. In most cases these patients do so without transfer letters. Our study documented a higher proportion of patients transferring out in the high perceived risk of HIV infection group compared to the low perceived risk group 17 (8.3) vs 17 (3.0).

Our study had several strengths and limitations. We reduced the potential for bias of our outcomes by administering the perceived risk for HIV infection question before testing for HIV. Participants self-reported details of perceived risk to HIV infection and HIV status prior to testing as part of our eligibility assessment for the study. Social desirability bias in their responses may result in an overestimation of the number of eligible participants. In our cohort, we did not analyse clinical factors which might affect rapid ART initiation besides CD4 count, and these are likely to be important confounders. We focused on demographic and social factors in this study.

## Conclusion

These findings suggest that social factors including perceived risk of HIV infection hinder optimal implementation of UTT. Persons with high perceived risk to HIV infection initiate ART more quickly compared to those with low perceived risk to HIV. We recommend extensive data on the acceptability of this strategy both by care providers and communities. There is need to intensify access to pre-exposure prophylaxis and empower individuals in low economic communities on all HIV prevention measures. To fill these gaps, studies are needed to identify additional potential barriers that can influence immediate ART initiation and retention in care. This can be achieved by conducting qualitative research that will aim to guide the design and implementation of interventions to improve ART initiation and retention in care. In addition, more investment is needed to increase awareness in communities about the benefits of ART to encourage same day HIV treatment initiation and reduce delays in HIV care linkage.

## Data Availability

The datasets used and/or analysed during the current study are available from the corresponding author on reasonable request.

## Abbreviations

ART: Anti-retroviral therapy
HIV: Human immunodeficiency viruses
AIDS: Acquired immunodeficiency syndrome
PLHIV: People living with HIV (PLHIV)
UNAIDS: Joint United Nations Programme on HIV/AIDS
WHO: World Health Organization
UTT: Universal test and treat
SPSS: Statistical Package for the Social Sciences
LTFU: Loss to follow up
